# The Importance of Genetic Screening on the Syndromes of Colorectal Cancer and Gastric Cancer: A 2024 Update

**DOI:** 10.3390/biomedicines12122655

**Published:** 2024-11-21

**Authors:** Iulia Lupan, Ciprian Silaghi, Claudia Stroe, Adriana Muntean, Diana Deleanu, Vasile Bintintan, Gabriel Samasca

**Affiliations:** 1 Department of Molecular Biology, Babes-Bolyai University, 400084 Cluj-Napoca, Romania; iulia.lupan@ubbcluj.ro; 2 Department of Biochemistry, Iuliu Hatieganu University of Medicine and Pharmacy, 400006 Cluj-Napoca, Romania; silaghi.ciprian@umfcluj.ro; 3 Department of Immunology, Iuliu Hatieganu University of Medicine and Pharmacy, 400006 Cluj-Napoca, Romania; claustroe@gmail.com (C.S.); adriana.muntean@umfcluj.ro (A.M.); deleanu@umfcluj.ro (D.D.); 4 Department of Surgery 1, Iuliu Hatieganu University of Medicine and Pharmacy, 400006 Cluj-Napoca, Romania; vasile.bintintan@umfcluj.ro

**Keywords:** genetics, colorectal cancer, gastric cancer

## Abstract

Gastrointestinal cancers (GIC), encompassing colonic, rectal, and gastric malignancies, rank among the most prevalent cancer types globally, contributing significantly to cancer-related mortality. In the scientific literature, various syndromes associated with colorectal and gastric cancers have been elucidated, highlighting the intricate interplay between genetic factors and disease manifestation. The primary objective of this study was to conduct a genetic exploration aimed at elucidating these associations and identifying shared genetic determinants across these cancer types. Notably, considerable research has focused on the KRAS gene mutations, polymorphisms in nucleic acids, the Wnt signaling pathway, and the role of chemokine ligands in tumorigenesis. While investigations into natural plant extracts as potential therapeutic agents are still in their nascent stages, they represent a promising avenue for future research. Ongoing studies are essential to uncover suitable biomarkers that could facilitate the identification and understanding of the genetic links between these GIC. This exploration not only seeks to enhance our comprehension of the underlying genetic architecture but also aims to inform the development of targeted therapies and preventive strategies.

## 1. Introduction

Gastrointestinal (GI) malignancies are characterized by significant heterogeneity at multiple levels, which poses challenges for diagnosis and treatment. Recent research has delved into tumor heterogeneity through molecular profiling techniques, including transcriptomic, proteomic, genetic, and epigenetic classifications. Among these, transcriptomic subtyping has emerged as a particularly advantageous approach, as it synthesizes information about the tumor microenvironment (TME), intrinsic cancer cell properties, and both genetic and epigenetic factors. This method has garnered attention due to its ability to reveal common biological characteristics across various GI tumors, which hold prognostic and predictive value. Notably, transcriptomic subgroups effectively capture intricate phenotypic states that can differ based on tumor location, therapeutic interventions, and the progression of the disease [1]. While advancements in diagnostic and management strategies have been made, the overall prognosis for patients with GI malignancies remains dishearteningly poor [2].

With an estimated 1.8 million new cases and over 881,000 deaths globally in 2018, CRC ranks as the second most prevalent cancer and the second leading cause of cancer mortality worldwide. The epidemiological landscape of CRC varies with respect to age, sex, race, and geographic factors, and is influenced by a myriad of elements, including risk exposure, demographic variations, genetic predisposition, and mutations impacting prognosis and treatment response [3]. According to the latest data from the World Cancer Research International Fund, approximately one million individuals globally were diagnosed with gastric cancer (GC) in 2020, underscoring the urgent need for the development of more effective tools for early detection, diagnosis, and prognostic assessment [4]. GC persists as a leading cause of cancer-related mortality worldwide. The incidence trends for this malignancy, however, are not static; epidemiological studies over the past decade indicate significant declines in GC rates among certain populations and demographic segments, contrasted with notable exceptions where rates are stable or increasing [5]. Furthermore, recent epidemiological data suggest a rising prevalence of various digestive system cancers, particularly among younger individuals—a phenomenon termed “early-onset cancer”. This trend is especially evident in colorectal cancer (CRC) and other malignant neoplasms affecting the digestive tract, particularly the stomach, with lesser involvement of the pancreas and biliary tract. Existing literature on digestive neoplasms is somewhat limited, focusing primarily on the stomach and colon [6]. The complexities surrounding genetic counseling, risk assessment, and clinical management of at-risk individuals and families are increasing, necessitating continuous education for healthcare professionals engaged in genetic testing, particularly in light of evolving data. The selection process for patients eligible for genetic testing is critical, hinging on familial cancer histories regarding type, number, and onset of malignancies [7]. Patients diagnosed with digestive malignancies frequently experience elevated levels of anxiety and depression, indicating that proactive clinical management of psychiatric disorders—via screening and preventive strategies—can enhance overall patient outcomes [8].

The prevalence of multiple primary tumors (MPTs), also known as second primary malignancies (SPMs), is a growing concern in the global oncology landscape. While estimates vary, recent studies highlight the significant incidence of this phenomenon. A 2017 report indicated a frequency of 2–17% for MPTs [9], while a 2022 study underscored the continual rise of SPMs in the Western world, with a 20% risk of a new primary cancer in patients with previously diagnosed carcinoma [10]. This trend holds particular significance in China, where, despite increasing survival rates for patients with MPTs, the quality of life remains subpar. Many patients present with advanced stage second primaries, hindering early intervention and effective treatment. This unfortunate reality is often attributed to misdiagnosis, with clinicians mistakenly classifying MPTs as metastases [11]. The escalating incidence of gastric and colorectal cancers further underscores the urgency for more robust research in the field of MPTs [12]. Larger sample sizes are crucial for gaining comprehensive insights that will ultimately inform refined clinical approaches to this complex dual malignancy. By fostering a deeper understanding of MPTs, clinicians can improve diagnosis and treatment, leading to improved outcomes and a higher quality of life for affected patients.

Furthermore, there is a critical need for routine surveillance for metachronous cancers during postoperative follow-up, particularly considering the influence of GC staging on prognostic outcomes, in contrast to CRC staging. Notably, dual syndromes associated with GC and CRC have been documented. Among these, two prominent hamartomatous polyposis syndromes, Peutz–Jeghers Syndrome (PJS) and juvenile polyposis syndrome (JPS), are particularly relevant. This category also encompasses other genetic conditions, such as Li–Fraumeni syndrome (LFS), Lynch syndrome (LS), familial adenomatous polyposis (FAP), and hereditary diffuse GC. A comprehensive assessment of these various syndromes and their implications is essential for the effective treatment of affected patients, necessitating an examination of genetic predisposition, cancer risk, known genetic aberrations, and recommended genetic testing [13].

In this study, we aim to investigate the genetic characteristics associated with CRC and GC, including genetic syndromes, other GI diseases with genetic implications, clinical risk factors, and the understanding of genetics in this context. Additionally, we will explore natural products as potential therapeutic options and identify prospective biomarkers. We performed a systematic assessment of the most relevant and recent literature in the PubMed database using the keywords “genetics”, “colorectal cancer”, and “gastric cancer”. We only looked at articles published between 2023 and 2024 and did not include case reports. 

## 2. Genetic Syndromes

GI neoplasms predominantly arise sporadically, yet there exists a notably elevated risk of developing GI tumors—both benign and malignant—associated with several well-established hereditary conditions. The advent of high-throughput molecular data has significantly enhanced our understanding of the pathophysiology underlying many of these disorders. Historically, some of these syndromes were characterized over a century ago based solely on clinical observations, without the benefit of modern molecular insights. In recent years, advancements in next-generation sequencing technologies have facilitated the identification of several high-risk genetic variants, offering new avenues for understanding these complex conditions. Despite the wealth of data available, substantial gaps in scientific knowledge remain, particularly regarding the implications of certain genetic variations. The management of these hereditary syndromes has undergone considerable transformation, largely due to increased access to genetic counseling and assessment for affected families. However, this progress has also led to significant psychological distress among patients, especially in instances where genetic variations present ambiguity [14].

LFS is a rare autosomal dominant hereditary cancer predisposition syndrome that arises from germline mutations in the TP53 gene, which encodes the p53 transcription factor, often referred to as the “guardian of the genome”. Individuals diagnosed with LFS exhibit an increased propensity for early-onset CRC and GC, as well as a heightened risk of developing multiple additional malignancies affecting various organs, including the skin, ovaries, thyroid, and lungs. Statistical analyses indicate that males with LFS face a lifetime cancer risk exceeding 70%, while females face an even more substantial risk, estimated at over 90%. To mitigate the potential for multiple primary malignancies throughout their lifetime, established screening protocols have been developed for individuals afflicted by LFS. Whole-body magnetic resonance imaging (MRI) is recommended as the primary modality for annual screening in this patient population [15]. The phenotypic manifestations resulting from germline pathogenic mutations in the TP53 gene exhibit variability influenced by factors, such as cell type, tissue specificity, age, and environmental context. Furthermore, the presence of frequent variants in patients who do not meet the clinical criteria for LFS underscores the necessity for enhanced predictive models that can effectively classify carriers into appropriate surveillance programs, thereby improving the accuracy of individual risk assessments [16]. Ongoing research is imperative to elucidate the clinical implications associated with the spectrum of germline TP53 mutations. The establishment of therapeutic subgroups within the LFS population may be warranted, predicated on the specific type of mutation present. Historical data reveal that earlier detection of malignancies in individuals carrying TP53 mutations correlates with diminished morbidity and enhanced survival outcomes [17]. Thus, a comprehensive understanding of the complexities associated with LFS and the role of TP53 mutations is crucial for optimizing patient management and improving prognostic indicators in affected individuals [18].

LS, commonly referred to as hereditary nonpolyposis CRC, represents the most prevalent hereditary form of CRC. This condition is associated with a variety of primary malignancies, including those affecting the stomach, ovaries, small intestine, prostate, biliary tract, pancreas, adrenocortical tract, and the urothelial tract (comprising the ureter, renal pelvis, and bladder). Additionally, LS has been linked to brain tumors, specifically glioblastomas, as well as sebaceous gland adenomas and keratoacanthomas [19]. The underlying etiology of LS is attributed to pathogenic variations that disrupt the function of mismatch repair (MMR) genes, which are crucial for maintaining genomic stability. In a recent study, researchers identified at-risk relatives by analyzing pedigrees of index patients. Among the 40 probands examined, the distribution of MMR gene mutations was observed as follows: MLH1 (67.5%), MSH2 (22.5%), MSH6 (7.5%), and PMS2 (2.5%). Notably, 58% of the 182 LS patients exhibited the LS phenotype prior to reaching the age of 50. CRC remains the most prevalent malignancy associated with LS, followed closely by GC in men and endometrial cancer in women [20]. Furthermore, a total of 242 paraffin-embedded tissue specimens from patients with GI, gynecological, genitourinary, lung, breast, and unidentified primary cancers were subjected to molecular analysis to determine microsatellite instability (MSI) status. This was accomplished through PCR-based molecular fragment analysis and immunohistochemistry. The literature indicates comparable detection rates for MSI-high patients identified via immunohistochemistry (23 patients) and those identified through molecular methods (29 patients). A strong concordance between the two diagnostic approaches was established (Kappa = 0.675 with a standard error of 0.081, *p* < 0.001). Given the findings, both immunohistochemistry and molecular analysis should be implemented as first-line screening tests for assessing MSI-H/dMMR status across all cancer types [21]. Additionally, the management of LS-associated malignancies may involve a multidisciplinary approach encompassing immunotherapy, chemotherapy, and vaccine-based interventions. However, there remains a pressing need for consensus on the optimal monitoring methodologies for these patients on a global scale [22].

Autosomal dominant polyposis syndrome, commonly referred to as FAP, represents a genetic condition characterized by varying levels of penetrance. The underlying genetic anomaly associated with FAP is a germline mutation in the adenomatous polyposis coli (APC) gene, which plays a critical role in the pathogenesis of the disorder. Clinically, FAP manifests through a spectrum of phenotypic variations, including but not limited to Gardner syndrome and Turcot syndrome. In the absence of timely intervention, affected individuals develop numerous polyps—ranging from hundreds to thousands—within the colon and rectum, typically emerging during early adolescence. The consequence of untreated FAP is an almost 100% lifetime risk of CRC, with the onset of malignancy frequently occurring by the age of 40. To mitigate the risk of CRC, prophylactic colectomy is often indicated. Furthermore, individuals diagnosed with FAP exhibit an increased susceptibility to various other malignancies, such as hepatoblastoma and desmoid tumors, as well as gastric and duodenal adenocarcinomas [23]. The clinical presentation of FAP patients with gastric lesions remains poorly understood. Notably, cumulative incidence rates for gastric adenoma and GC among 50-year-old individuals with FAP are reported at 22.8% and 7.6%, respectively. Interestingly, no significant correlation has been established between the colonic phenotype of FAP and the presence of gastric neoplasms. The hazard ratio (HR) for the development of gastric adenomas peaks around the age of 65, reaching an HR maximum of 0.043. Conversely, the HR for GC demonstrates a progressive increase until approximately age 40, after which there is a marked escalation (HR = 0.0067). Given these findings, it is essential to implement thorough surveillance of the upper GI tract in older FAP patients, potentially adjusting follow-up intervals based on age to facilitate early detection of GC [24]. Effective management of FAP necessitates a multidisciplinary and individualized approach. Enhancements in patient outcomes and quality of life can be achieved through the integration of genetic advancements, innovative surveillance techniques, and newly emerging therapeutic modalities [25]. However, it is noteworthy that patient compliance, particularly concerning thyroid ultrasonography, remains suboptimal. This underscores the critical importance of patient education and health literacy in managing FAP. Healthcare providers must emphasize the significance of genetic counseling and routine surveillance to ensure comprehensive care for patients affected by this syndrome [26].

JPS is classified as an autosomal dominant genetic disorder characterized by an increased risk of GIC and a predisposition to the formation of juvenile polyps throughout the GI tract [27]. This condition exhibits notable phenotypic diversity, particularly in the localization of polyps, malignancy risk, and the presence of extraintestinal symptoms among individuals carrying mutations in either the BMPR1A or SMAD4 genes. It is important to note that researchers face challenges in determining the specific mutation locations within the BMPR1A gene based solely on phenotypic characteristics. Nevertheless, the phenotypic traits associated with BMPR1A genetic variant carriers predominantly correlate with polyps found in the colon and rectum, which may aid in assessing the potential pathogenicity of these variations [28]. Currently recognized causal genes for JPS include SMAD4 and BMPR1A. Epidemiological data indicate that approximately 25% of newly diagnosed JPS cases are sporadic, lacking a familial history of polyposis, while the remaining 75% are attributed to an autosomal dominant inheritance pattern. Furthermore, many patients with JPS develop GI lesions during childhood, which can persist into adulthood, necessitating continuous medical surveillance. Historically, the polyps associated with JPS were acknowledged for their potential malignancy; however, they are primarily classified as hamartomatous polyps, exhibiting a low risk of carcinogenesis. Notably, certain polyps in individuals with JPS may progress to adenomas, which can subsequently trigger carcinogenic processes. In other instances, polyps may evolve into carcinomas independently of adenoma formation. Currently, there is no curative treatment available for JPS. Surgical intervention is recommended in cases where the patient experiences intestinal intussusception, invasive cancer, hypoproteinemia, refractory anemia, or when endoscopic resection is indicated. Specifically, endoscopic resection is advised for symptomatic polyps to alleviate associated complications and enhance patient outcomes. Continued research is essential to further elucidate the genetic mechanisms underlying JPS and to develop more effective management strategies for affected individuals [29].

PJS is characterized by the presence of GI polyps, mucocutaneous pigmentation, and an elevated risk of malignancies. The presence of specific genetic variants in the STK11 gene is the primary etiological factor contributing to this syndrome [30]. While individuals without mutations in STK11 exhibit a reduced risk of developing cancer and typically experience a later onset of symptoms, those with the uncommon splicing mutation c.921-1G > C in intron 7 of STK11 face significant health challenges due to its detrimental effects. The c.921-1G > C mutation has been identified as particularly harmful, despite its location in intron 7, which raises questions about its impact on STK11 expression. This splicing mutation may not lead to the production of aberrant transcripts, specifically the deletion of 40 base pairs between exons 3 and 4; however, it is still associated with a notable reduction in STK11 expression levels. The implications of such genetic variations are profound, necessitating advanced strategies for early complication management. The management of PJS emphasizes the importance of presymptomatic surveillance, genetic counseling, and early intervention strategies. This is especially crucial considering the risks associated with polyps in children diagnosed with PJS, who may require multiple laparotomies due to complications. The condition often leads to diffuse involvement of the intestines, complicating surgical interventions and necessitating cautious decision-making regarding resection. Conservative measures should be prioritized, especially when surgical concerns arise. In instances of intussusception, the recommended approach involves limited resection or polypectomy following a decrease in the incidence of the condition [31]. The clinical presentation of PJS may exhibit unique characteristics among Russian patients, indicating the need for customized guidelines for therapy and monitoring [32]. These distinctions highlight the importance of understanding genetic, environmental, and cultural factors that contribute to the variability in disease progression among different populations. In summary, the management of PJS requires a multifaceted approach that considers the specific genetic variants of the STK11 gene and their implications for cancer risk and clinical outcomes. Early intervention, vigilant surveillance, and tailored treatment strategies are essential for improving the quality of life and health outcomes for patients with PJS. Continued research and development of protocols that address the unique challenges faced by diverse populations will further enhance the care of individuals affected by this syndrome [33].

Recognizing GI tumors that manifest within familial contexts is of paramount importance in the field of oncology and genetic research. These tumors, often exhibiting hereditary patterns, necessitate heightened awareness and understanding due to their implications for both individual and familial health. The identification of such tumors not only aids in early diagnosis and intervention but also contributes to the broader discourse on genetic predispositions to cancer.

There exists a compelling need for a differential diagnostic procedure that is verifiable through germline genetic analysis. The precise identification of germline syndromes is critical, as it facilitates the implementation of tailored surveillance and care protocols guided by relevant, condition-specific clinical guidelines. Such an approach necessitates a comprehensive understanding of various predisposing factors associated with GI and extraintestinal malignancies. As outlined in previous research [34], recognizing these factors is essential for optimizing patient outcomes and informing clinical decision-making in the context of hereditary cancer syndromes.

## 3. Other GI Diseases with Genetic Implications

Initial p53 mutations are crucial in the “chronic inflammation-dysplasia-cancer” pathway of carcinogenesis, which is closely related to the development of colitis-associated colorectal carcinoma (CAC) in the context of inflammatory bowel disease (IBD). This pathological progression begins with gastric metaplasia (GM), which signifies the first step in the serrated CRC development and is induced by persistent stress on the colonic mucosa. Within the serrated pathway, GM is particularly evident in the inflammatory mucosa of IBD patients and can persist in individuals experiencing long-term alterations. Notably, GM disappears upon the acquisition of p53 mutations, indicating a pivotal transition in the disease process [35]. Recent studies have identified three specific methylation sites that prove instrumental in distinguishing between sporadic carcinoma (sCRC) and CAC. When methylation values at these sites are specific, they effectively classify samples as either CAC or sCRC based on predetermined methylation limit values. Remarkably, this triad of methylation markers allows for accurate classification in 94.5% of cases, showcasing their potential utility in the differential diagnosis of CAC versus sCRC [36]. However, the limitations of existing surveillance techniques become apparent, particularly when considering the significant proportion of cases that appear to arise from non-adenomatous mucosa. This type of mucosa is characterized by rapid cancer progression, yet it often fails to exhibit neoplasia upon biopsy [37]. Recent investigations have shed light on new therapeutic avenues, particularly the development of a stimulator of interferon genes (STING) as a target for ulcerative colitis (UC) and CAC. This is supported by findings that demonstrate the detection of the TFAM-mtDNA complex from the damaged intestinal epithelium by myeloid STING, which exacerbates colitis through the action of interleukin-12 (IL-12) cytokines. These insights underscore the complex interplay between inflammation, genetic mutations, and potential therapeutic targets in the management of CAC within the IBD patient population [38].

GI stromal tumors (GISTs) represent a unique and uncommon category of neoplasms originating in the GI tract, characterized by the presence of spindle, epithelioid, or occasionally pleomorphic cells. These tumors are derived from interstitial cells of Cajal, which serve as critical pacemakers for GI motility. The pathophysiology of GISTs is closely linked to mutations in the c-kit and PDGFRA genes, alterations that are frequently identified through immunohistochemical labeling. Epidemiological data indicate that the stomach is the most frequently affected organ, accounting for 35% of cases (n = 7), followed by the colon (15%, n = 3) and peritoneum (5%, n = 1), with a total of 45% (n = 9) of tumors localized to these sites. Histopathological evaluation reveals a spectrum of tumor types: 10% (n = 2) of cases are classified as epithelioid tumors, 50% (n = 10) as mixed tumors, and 40% (n = 8) as spindle-type tumors. The prognosis for patients diagnosed with GISTs is predominantly influenced by tumor size and the mitotic index, highlighting the importance of early detection and intervention [39]. Recent advancements in molecular diagnostics, such as digital droplet polymerase chain reaction (ddPCR) testing, have identified the PDGFRA D842V mutation within GIST samples. However, the low concentration of circulating DNA in plasma specimens presents challenges for early-stage mutation detection, underscoring the need for robust biomarker identification methods. The area under the curve (AUC) analysis of the hypermethylated SEPT9 gene has yielded promising diagnostic performance metrics, with AUC values of 0.74 (95% confidence interval (CI): 0.52–0.97), 0.77 (95% CI: 0.56–0.98), and 0.79 (95% CI: 0.59–0.99). Given the rarity of GISTs, the timely identification of these tumors using specific biomarkers is crucial for improving patient outcomes [40]. Moreover, imaging modalities, such as [18F] FAPI-42 PET/CT and [18F] FDG PET/CT, are valuable tools for predicting treatment responses in recurrent or metastatic GISTs. The integration of these imaging techniques with genetic mutation data and targeted therapy information allows for the development of nomograms that enhance the accuracy of prognostic evaluations, ultimately facilitating personalized treatment approaches for patients with GISTs. In conclusion, a comprehensive understanding of GISTs, encompassing their biological behavior, genetic underpinnings, and advancements in diagnostic strategies, is essential for clinicians aiming to optimize patient management and improve long-term outcomes. Continued research and refinement of diagnostic tools will play a pivotal role in the early detection and effective treatment of this rare malignancy [41].

Table 1 provides a comprehensive overview of the primary syndromes associated with GC and CRC. This summary serves as a valuable reference for understanding the interrelations between these malignancies and their corresponding clinical syndromes. By delineating the key syndromes linked to GC and CRC, the table facilitates a clearer understanding of the potential risk factors, genetic predispositions, and phenotypic presentations that may influence patient outcomes. Additionally, this synthesis of information underscores the importance of early detection and tailored interventions in managing these complex conditions. The insights derived from Table 1 can enhance the clinical acumen of healthcare professionals and ensure a more informed approach to the diagnosis and treatment of patients at risk of GC and CRC.

## 4. Genetic Mutations and Pathways in Gastric and Colorectal Adenocarcinomas: Implications for Carcinogenesis and Therapeutic Strategies

### 4.1. KRAS Gene

Gastric and colorectal adenocarcinomas represent significant challenges in oncology due to their high prevalence and critical role in carcinogenesis and cancer progression. Mutations in key oncogenes and tumor suppressor genes, such as p53, RAS, and MDM2, have been extensively documented in these malignancies. In a recent study, genomic DNA was isolated from 200 gastric tissue samples and 233 colon and rectal adenocarcinoma samples to investigate these mutations. Notably, deletions in exons 5–6 of p53 and mutations in HRAS at codons 12 and 61 were identified in 78% of poorly differentiated adenocarcinomas. Furthermore, p53 exon–intron 7–9 deletions were detected in 50–60% of adjacent tissues and 100% of moderately differentiated adenocarcinomas. The loss of WAF1 gene expression was observed in 20% of neighboring tissue samples and in nearly 90% of poorly differentiated adenocarcinomas, indicating a potential mechanism for tumor progression. The study also highlighted that approximately 63.9% of colon and rectal samples exhibited mutations in KRAS and NRAS, suggesting a propensity for autonomous cell proliferation [42]. KRAS, the most frequently mutated oncogene in human cancers, generates two isoforms—KRAS4a and KRAS4b—through alternative splicing. Distinct differences between these isoforms, particularly in exon 4, which encodes critical regions for membrane localization and trafficking, were analyzed. The thermal stability of KRAS4a is notably influenced by amino acid position 151, while position 153 affects its binding affinity for RAF1 CRD protein. In the stomach, liver, and bile ducts, KRAS4a transcript levels were significantly higher than those of KRAS4b, whereas in the colon and rectum, both isoforms exhibited comparable expression levels [43]. Understanding the frequency and variability of KRAS alleles in CRC patients is crucial for optimizing individualized therapeutic approaches and enhancing the overall comprehension of the disease’s genetic landscape [44]. Additionally, the expression of GFRA3 in GC has been correlated with the activation of two cancer-associated pathways: the KRAS signaling pathway and epithelial–mesenchymal transition (EMT). Gene set enrichment analysis revealed that GFRA3 not only induces EMT markers but also activates the PI3K/AKT pathway and extracellular signal-regulated kinase (ERK), consequently enhancing the migration and invasion capabilities of GC cells via KRAS-mediated signaling. Furthermore, ARTN facilitates the EMT, migration, and invasion of GC cells through GFRA3, with the KRAS inhibitor demonstrating a reduction in the effects of the ARTN-GFRA3 axis [45]. In summary, this investigation underscores the critical role of genetic mutations and signaling pathways in the progression of gastric and colorectal adenocarcinomas. It emphasizes the need for further exploration of KRAS mutations and their implications for personalized treatment strategies, ultimately aiming to improve patient outcomes in these challenging malignancies.

### 4.2. Wnt Signalling Pathway

The oncogenic transcription factor STAT3 plays a crucial role in the development and progression of these tumors. STAT3, often overactivated in human cancers, including GI tumors, stimulates the transcription of genes involved in metastasis, anti-apoptosis, proliferation, and survival, thus accelerating tumor growth, metastasis, and drug resistance. Recent research has shed light on the complex interplay between STAT3 and other signaling pathways, particularly the Wnt pathway, in driving GI tumorigenesis [46]. The Wnt receptor ROR1 has emerged as a promising therapeutic target in various cancers. Studies have demonstrated a high frequency of ROR1 expression in several tumor types, including mesothelioma, liposarcoma, GISTs, and uterine endometrioid carcinoma. This finding underscores the potential of targeting ROR1 for the treatment of these cancers [47]. Furthermore, the identification of Wnt-regulated long noncoding RNAs (lncRNAs), such as CCAT5 and CCAT2, has revealed their critical roles in regulating STAT3 activity and promoting tumor development. Specifically, CCAT5 interacts with STAT3, preventing its dephosphorylation and nuclear translocation, thereby accelerating GC progression [48]. Similarly, CCAT2, through its protein-binding properties, influences the alternative splicing of CD44, promoting the progression of GC [49]. The involvement of LBH, a developmental transcription cofactor, further highlights the complexity of the Wnt pathway in cancer. LBH overexpression, observed in a broad spectrum of malignancies, including those affecting the GI tract, is associated with a poor prognosis and suggests its potential as a biomarker for Wnt hyperactivation in cancer patients. These findings highlight the intricate molecular mechanisms underlying GI tumorigenesis, emphasizing the importance of understanding the interplay between STAT3, the Wnt pathway, and lncRNAs [50]. Further research focused on these pathways may lead to the development of novel therapies aimed at disrupting these critical signaling nodes, ultimately improving the treatment of GIC.

### 4.3. Nucleic Acid Polymorphisms

The pathogenesis of GC and intestinal cancer, also known as CRC, is a complex interplay of genetic and epigenetic factors. A study investigated the role of specific gene polymorphisms and RNA modifications in the development and progression of these malignancies. The research employed a PCR-RFLP approach to analyze the genotypes of genes associated with GC and CRC in 200 healthy individuals and 200 cancer patients. The results revealed a significant association between the rs10811661 polymorphism in the CDKN2A/B gene and the development of both GC and CRC. This polymorphism has been previously linked to tumor invasiveness and size [51]. Another study further explored the role of RNA modifications, specifically N6-methyladenosine (m6A) and N7-methylguanosine (m7G), in the regulation of gene expression. The methylation of RNA by METTL1 and METTL3 was shown to influence the expression of key cell cycle regulators, such as CDK4. In particular, METTL3-mediated METTL1 upregulation through m7G modification of CDK4 was found to enhance cell proliferation in head and neck squamous cell carcinoma (HNSC) [52]. Additionally, a study observed a correlation between METTL3 expression and the splicing factor SRSF11 in several cancer types, including breast invasive ductal carcinoma, lung adenocarcinoma, CRC, and GC. METTL3-mediated downregulation of SRSF11 expression was associated with poor prognosis in these cancers. These findings highlight the crucial roles of both genetic and epigenetic dysregulation in the development and progression of GC and CRC. The rs10811661 polymorphism in the CDKN2A/B gene represents a potential genetic susceptibility factor, while RNA modifications, particularly m7G and m6A, contribute to the aberrant regulation of key cell cycle and splicing proteins. Further research is needed to elucidate the complex interactions between these genetic and epigenetic factors and their implications for therapeutic interventions.

Recent research has highlighted the significant role of CDK20 (cyclin-dependent kinase 20) and MGMT (O6-methylguanine-DNA-methyltransferase) in the development and treatment of various cancers. CDK20, previously known as CCRK (cell cycle-related kinase), acts as a key regulator of cell cycle checkpoints. Its dysregulation has been observed in a range of malignancies including ovarian, brain, colon, gastric, liver, and lung cancers. CDK20’s involvement in the EZH2/NF-κB, KEAP1-NRF2, and Wnt signaling pathways further underscores its critical role in cancer progression [53]. MGMT, on the other hand, is an enzyme responsible for DNA repair, specifically by removing alkylating agents that can damage DNA and lead to tumor formation. Its activity is influenced by genetic polymorphisms, methylation, and protein expression, factors that impact tumor development and patient prognosis in various cancers, including gastric (GC) and colorectal (CRC) cancers. Understanding the intricate interplay between CDK20 and MGMT provides valuable insights for developing targeted therapies. Current strategies focus on three main approaches: (1) immunotherapy in conjunction with alkylating agents to target tumors, (2) targeting MGMT to enhance tumor sensitivity to alkylating chemotherapy, and (3) treating patients with nonmethylation of the MGMT promoter. This research highlights the critical need for further investigation into the specific mechanisms by which CDK20 and MGMT influence cancer development and treatment to optimize therapeutic strategies and improve patient outcomes [54].

### 4.4. Chemokine Ligands

While extensive studies have explored the general involvement of chemokines in cancer development, a comprehensive understanding of the specific role of CXCL1, also known as MGSA or GRO-α, remains elusive [55]. This chemokine, along with its six counterparts (CXCL2, CXCL3, CXCL5, CXCL6, CXCL7, and CXCL8/IL-8), constitutes the ligands for the CXCR2 receptor, all exhibiting similar properties. This study investigates the multifaceted role of CXCR2 ligands across 31 distinct cancer types, encompassing glioblastoma, melanoma, and various GI, renal, hepatic, pulmonary, gynecological, pancreatic, and prostate cancers. Leveraging bioinformatic analyses through the GEPIA, UALCAN, and TIMER2.0 databases, the study reveals a complex interplay between CXCR2 ligands and cancer prognosis, dependent on the specific cancer type. Furthermore, the study identifies a strong correlation between CXCR2 ligands and key processes involved in cancer development, including epithelial-mesenchymal transition (EMT), angiogenesis, neutrophil infiltration into the TME, and M1 macrophage abundance. Importantly, the study highlights the unique expression patterns and participation of individual CXCR2 ligands in cancer progression, emphasizing the need for a nuanced understanding of their individual roles [56]. Finally, another study presents a novel CRC risk model based on six genes (ZG16, MPC1, RBM47, SMOX, CPM, and DNASE1L3), with RBM47 identified as a prognosis-related gene linked to CXCL13 in the TLS region. This finding underscores the significant potential of CXCL1 and its associated genes in improving CRC prognosis and developing targeted therapeutic approaches [57].

### 4.5. Other Genes

Recent advances in genomic research have shed light on the multifaceted role of glutamine in GI health. A genome-wide association study (GWAS) involving 114,751 participants identified genetic instrumental variables for glutamine exposure, revealing significant associations between blood glutamine levels and various GI disorders. Specifically, the study reported an inverse relationship between glutamine levels and the risk of CRC (odds ratio [OR] = 0.998; 95% CI: 0.997–1.000; *p* = 0.027), colitis (OR = 0.998; 95% CI: 0.997–1.000; *p* = 0.020), and IBD (OR = 0.551; 95% CI: 0.343–0.886; *p* = 0.014). Conversely, no substantial association was observed between glutamine levels and gastroesophageal cancers (GCs) (OR = 0.966; 95% CI: 0.832–1.121; *p* = 0.648). These findings underscore the potential protective effects of glutamine in European populations against colitis, Crohn’s disease, CRC, and IBD [58]. Moreover, the therapeutic potential of glutamine supplementation in conjunction with exercise has been evaluated in animal models. Research focused on rats with UC demonstrated that interventions involving strength and endurance training, paired with L-glutamine administration, led to marked improvements in cytokine profiles and oxidative stress levels, despite persistent stomach dysmotility. These results suggest that such combined interventions may effectively mitigate intestinal inflammation. The evidence gathered from both genomic studies and animal models points to glutamine as a crucial factor in the prevention and management of GI disorders. Future research should explore the mechanisms underlying glutamine’s protective effects and the clinical implications of these findings for dietary and therapeutic interventions in patients with GI diseases [59].

Other research delves into the potential therapeutic benefits of inhibiting taurine (SLC6A6) and creatine (SLC6A8) transporters, while also investigating their possible involvement in cancer development. Experimental data suggest a correlation between overexpression of these proteins and malignancies, particularly in colon and breast cancers, two of the most prevalent cancer types [60]. The focus on the taurine transporter (TauT, SLC6A6) stems from its crucial role in various physiological processes and its observed overexpression in GIC and CRC progression. Utilizing homology modelling techniques, TauT models were generated and analyzed through molecular dynamics (MD) simulations and docking investigations. These in silico studies, alongside comparisons with other GABA transporter group members, revealed the critical role of three amino acids—Glu406, Gly62, and Tyr138—in ligand binding. Further structural analysis of TauT mutants confirmed the significance of these specific residues. The findings support the development of novel taurine transporter inhibitors as potential anticancer agents [61]. This research highlights the promising therapeutic implications of targeting these transporter systems, presenting a potential avenue for future cancer treatment development.

The complex interplay between the immune system and cancer is a critical area of ongoing research, with significant implications for the development of effective therapies. One promising target for immunotherapeutic strategies is the interaction between the sushi domain-containing protein 2 (SUSD2) and interleukin (IL)-2 receptors. This interaction, as demonstrated in [62], negatively impacts the activity of antitumoral CD8+ T-cells, a crucial component of the immune response against cancer. SUSD2, also known as the complement control protein domain, plays a multifaceted role in various physiological and pathological processes. Notably, it exhibits contrasting effects on different tumor types, acting as a tumor suppressor in lung, bladder, and colon cancers while promoting tumorigenesis in breast, gastric, and glioma cancers [63]. This dual role highlights the intricate relationship between SUSD2 and cancer development. The regulation of SUSD2 expression is equally complex, involving a diverse array of factors. Noncoding RNAs, methylation of its promoter, and other molecules, like p63, tropomyosin alpha-4 chain, and galectin-1, all contribute to the fine-tuning of SUSD2 expression [64]. Understanding the precise mechanisms governing SUSD2 regulation is crucial for developing targeted therapeutic strategies aimed at manipulating its activity and ultimately influencing the course of cancer progression. Further research into the SUSD2-IL-2 receptor axis holds significant potential for advancing immunotherapy strategies. By unraveling the intricate molecular mechanisms governing this interaction, we may be able to develop novel approaches that enhance antitumoral CD8+ T-cell activity, potentially leading to improved outcomes for cancer patients.

While certain proteins, like EMILIN-1, exhibit antiproliferative effects in skin cancer and CRC, their function becomes complex in GC. EMILIN-1 contributes to lymphatic dysfunction and promotes proliferation, thereby supporting carcinogenesis. This highlights the tissue-specific nature of protein function and the potential for a protein to act as a tumor suppressor in one context and an oncogene in another [65]. Further complicating the picture is the interplay between various genes and proteins. For instance, DARS, encoding aspartyl-tRNA synthetase, has been implicated in the pathogenesis of multiple cancers, including GC, colon cancer, glioblastoma, and renal cell carcinoma [66]. Similarly, NTRK3, a neurotrophic receptor tyrosine kinase, functions as a tumor suppressor in CRC and neuroblastomas but acts as an oncogene in gastric and breast cancers. The complex interplay extends beyond individual proteins [67]. Increased expression of TMEM120B, a transmembrane protein, has been observed in various cancers, including lung, breast, stomach, colon, and ovary, and correlates with lymph node metastasis, advanced stage, and poor prognosis [68]. Additionally, the Krebs cycle enzymes, namely fumarate hydratase and citrate synthase, promote aerobic glycolysis and cancer cell proliferation, while MAEL plays an oncogenic role in stomach, colon, liver, and bladder cancers [69]. Interestingly, the transcription factor SOX17, crucial for cell differentiation and development, is absent in several cancers, including cervical squamous cell carcinoma, mesothelioma, and carcinomas of the breast, lung, pancreas, colon, stomach, liver, bladder, and salivary glands. While cervical adenocarcinoma displays low SOX17 expression, suggesting its potential role as a tumor suppressor, the intricate relationship between genes, proteins, and cancer development is far from fully understood. This review highlights the need for further research to elucidate the specific mechanisms by which these molecules contribute to cancer initiation, progression, and metastasis, paving the way for the development of novel, targeted therapies [70].

**Figure 1:**
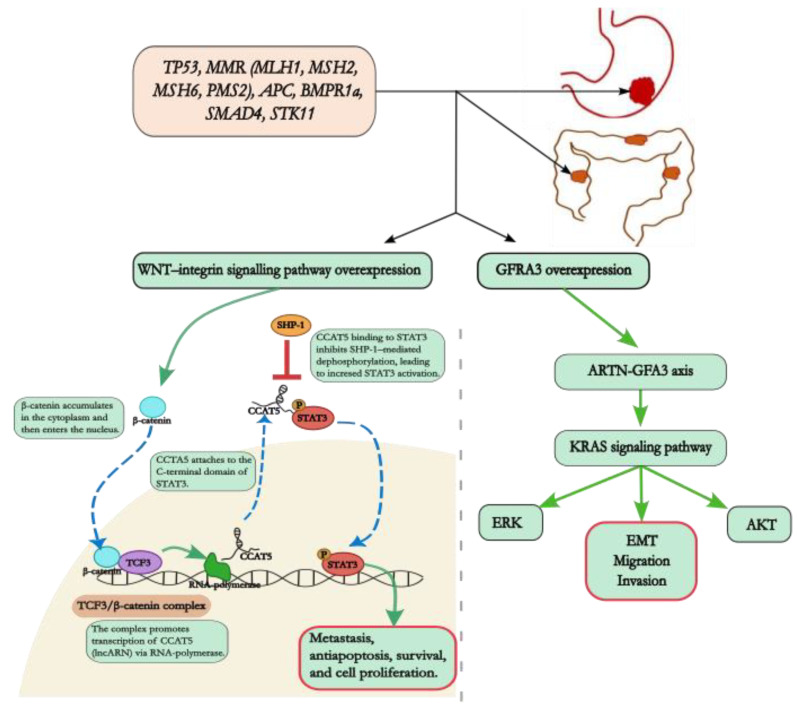
Genes commonly associated with tumorigenesis in CRC and GC, as well as important mechanisms involved in cancer development and progression.

As illustrated in Figure 1, the familial occurrence of GI tumors underscores the critical need for thorough screening and genetic counseling, particularly for individuals with a documented history of these malignancies in their families. By enhancing our recognition of these tumors, we can improve patient outcomes and implement more effective preventive strategies.

## 5. Natural Products as Possible Treatment Options

A phytochemical with promising anticancer potential is arctiin, a naturally occurring lignan found in various plants, and it has garnered significant attention for its diverse pharmacological properties. Traditionally utilized as a vegetable in Asian cuisine and as an ingredient in European culinary practices, arctiin has demonstrated a range of biological activities, including antibacterial, antitumor, antioxidative, antiproliferative, and antisenescence effects [71]. Notably, its anticancer properties have been extensively investigated in preclinical studies, revealing its potential as a therapeutic agent against various malignancies. Numerous studies have highlighted the potent anticancer effects of arctiin on tumor growth, exhibiting efficacy against a spectrum of cancers, including cervical, myeloma, prostate, endothelial, gastric, and colon cancers. The mechanisms underlying these effects are multifaceted and involve the modulation of key cellular pathways. Arctiin has been shown to induce mitochondrial dysfunction, interfere with cell cycle progression, particularly at the G2/M phase, inhibit cell proliferation, promote apoptotic cell death, exert cytotoxic effects, and suppress the migration and invasion of malignant cells. These findings suggest that arctiin’s anticancer activity is mediated by its ability to target multiple cellular processes, thus impeding tumor growth and development [72]. Further research is warranted to elucidate the precise mechanisms of action and optimize the therapeutic potential of arctiin for clinical applications. However, the existing evidence strongly suggests that arctiin holds significant promise as a novel anticancer agent, warranting further exploration and investigation in the fight against cancer.

While conventional therapies have proven effective in some cases, the development of drug resistance remains a significant hurdle. This has fueled interest in exploring alternative and complementary approaches, including natural compounds with anticancer properties. One such compound of interest is noscapine, a phthalide isoquinoline alkaloid derived from the opium poppy [73]. Noscapine has demonstrated anticancer activity against a range of cancer types, including gastric, ovarian, colon, and breast cancers. Its mechanism of action involves the induction of apoptosis, a process of programmed cell death essential for the control of cancer cell growth. Research has shown that noscapine influences the expression of key genes and proteins involved in the apoptotic pathway, including RELA, CASP8, CASP9, NFKBIA, and BAX, in human breast cancer (BC) cell lines (MCF-7 and MDA-MB-231). This suggests that noscapine can effectively target and eliminate cancer cells through apoptosis. However, several cancer types exhibit resistance to the anticancer effects of noscapine and its derivative, cotarnine, a tetrahydroisoquinoline (THIQ) scaffold created by the oxidative degradation of noscapine. To overcome this challenge, researchers are exploring novel strategies, including the development of amino acid conjugates of noscapine and cotarnine. This derivatization approach holds promise for enhancing the anticancer activity of these compounds, potentially improving their therapeutic efficacy and circumventing resistance mechanisms [74]. Further research is necessary to fully understand the potential of noscapine, cotarnine, and their amino acid conjugates in cancer treatment. However, the available evidence suggests that these compounds offer promising avenues for developing new, effective, and potentially less toxic cancer therapies.

Guggulsterone (GS), a phytosterol derived from the gum resin of the Commiphora mukul tree, has garnered significant attention in recent scientific research for its potential therapeutic applications in cancer treatment. Investigations into the action mechanisms of GS have revealed its impact on key survival pathways that are often constitutively activated in cancer cells. Specifically, studies have focused on the nuclear factor-kappa B (NF-kB), phosphoinositide 3-kinase/AKT (PI3K/AKT), and Janus kinase/signal transducer and activator of transcription (JAK/STAT) signaling pathways. These pathways play critical roles in regulating proinflammatory and antiapoptotic gene expression, which are fundamental to the inflammatory response and cellular growth. The interaction of GS with these survival pathways suggests its potential to disrupt the mechanisms that confer resilience to cancer cells [75]. Notably, the inhibition of the NF-kB, PI3K/AKT, and JAK/STAT pathways positions GS as a promising candidate for cancer therapy. Empirical evidence supports the notion that GS exhibits both curative and prophylactic effects against various cancer types. Its multifaceted actions—including the induction of apoptosis, antiangiogenic effects, and the modulation of diverse signaling cascades—underscore its capability to inhibit tumor growth and promote tumor regression. By triggering apoptotic pathways, GS effectively diminishes the viability of certain cancer cells, further solidifying its role as a potential therapeutic agent in oncology [76]. Thus, the exploration of GS as a viable option for cancer treatment not only highlights its pharmacological significance but also underscores the need for further research to fully elucidate its mechanisms of action and therapeutic applications.

Resveratrol, a phytoalexin polyphenolic compound, is found in a variety of dietary sources, including cereals, peanuts, grapes, strawberries, and raspberries. This naturally occurring compound has garnered significant attention for its potential protective effects against various cancers and cardiovascular diseases (CVD) [77]. The therapeutic efficacy of resveratrol can be attributed to its anti-inflammatory and anticancer properties, which are mediated through the regulation of long noncoding RNAs (lncRNAs). Research indicates that resveratrol functions as an anticancer agent by modulating both tumor-supportive and tumor-suppressive lncRNAs. Specifically, it downregulates the expression of several oncogenic lncRNAs, including DANCR, MALAT1, CCAT1, CRNDE, HOTAIR, PCAT1, PVT1, SNHG16, AK001796, DIO3OS, and H19, while simultaneously upregulating tumor-suppressive lncRNAs, such as MEG3, PTTG3P, BISPR, PCAT29, GAS5, LOC146880, PCA3, and NBR2. This regulatory interplay leads to the induction of apoptosis and increased cytotoxicity in cancer cells, presenting resveratrol as a promising candidate for cancer therapy. To fully harness the potential of polyphenols, like resveratrol, in cancer treatment, a deeper understanding of the mechanisms underlying lncRNA modulation is essential [78]. Future investigations should focus on elucidating these pathways to enhance the efficacy of resveratrol in clinical settings and to improve therapeutic outcomes for patients suffering from cancer.

In the context of cancer pathophysiology, garcinol has emerged as a compound of significant interest due to its multifaceted biological activities. This polyisoprenylated benzophenone, derived from various species of the Garcinia genus, exhibits notable antioxidant, anti-inflammatory, and histone acetyltransferase (HAT) inhibition properties, alongside its role as a microRNA (miRNA) deregulator. Recent research has demonstrated that garcinol effectively inhibits all three examined hyaluronidase enzymes, with findings indicating that this inhibition is both reversible and noncompetitive [79]. Molecular docking studies further elucidate the mechanism of action, revealing that hydrophobic interactions and hydrogen bonding facilitate the binding of garcinol to the hyaluronidase enzyme. The therapeutic potential of garcinol has garnered attention for its application in preventing GIC. Specifically, studies suggest that garcinol plays a crucial role in inhibiting metastasis, inducing apoptosis, and targeting pivotal molecular pathways involved in cancer progression. Its anticancer efficacy has been documented across various malignancies, including pancreatic, liver, esophageal, gastric, and colorectal cancers. Importantly, the safety evaluations conducted thus far indicate a promising toxicity profile for garcinol, suggesting its viability as a natural therapeutic agent for GI malignancies. However, despite these encouraging findings, further research is essential to confirm garcinol’s safety and efficacy in clinical settings. Future investigations should focus on optimizing its pharmacokinetics and exploring potential synergistic combinations with other therapeutic agents [80]. By doing so, we may unlock the full potential of garcinol as a viable treatment option in oncology, particularly for patients afflicted with GIC.

Minerals, particularly trace elements, play a crucial role as micronutrients that are essential for maintaining the body’s normal physiological functions. Various minerals exert different effects on cancer states, with their impact being influenced by such factors as the tumor’s location and the availability of specific minerals, like calcium. For instance, zinc is noted for its ability to enhance immune system functionality and facilitate wound healing, thereby contributing positively to patient outcomes. On the other hand, selenium exhibits a dose–response relationship with various types of cancer and is recognized for its antioxidant properties, which may help mitigate oxidative stress associated with malignancies [81]. Moreover, vitamins, as powerful antioxidants, have the potential to significantly lower cancer risk by neutralizing free radicals that can lead to cellular damage. In certain circumstances, nutritional supplements that are tailored to an individual’s genetic profile, dietary habits, tumor histology, and treatment regimen may offer substantial benefits. A poorly balanced diet can compromise the immune system and diminish treatment tolerance, ultimately reducing the efficacy of chemotherapy in eradicating malignant cells. Presently, a considerable proportion of cancer patients utilize vitamin supplementation to augment standard medical care and alleviate the adverse effects associated with both their treatments and the disease itself [82]. As such, the integration of nutritional strategies into cancer care regimens represents a promising avenue for enhancing patient outcomes and improving overall quality of life.

Table 2 presents a comprehensive summary of the effects of natural products as potential treatment options. This table systematically categorizes various natural substances and their observed pharmacological activities, highlighting their efficacy in addressing specific health conditions. The data compiled in this table are crucial for researchers and practitioners alike, as they provide insight into the therapeutic potential of these natural products, alongside relevant dosage information and potential side effects. The synthesis of this information fosters a deeper understanding of alternative and complementary therapies, paving the way for further exploration and integration into holistic treatment paradigms.

## 6. Possible Biomarkers

In the pursuit of enhanced cancer detection methodologies, the plasma cell-free DNA (cfDNA)-based approach known as TOTEM has emerged as a significant innovation, particularly in the identification and tracking of cancer signals through methylation markers. This method involved targeted methylation sequencing facilitated by enzymatic conversion, applied to plasma cfDNA samples collected from a substantial clinical cohort consisting of 733 cancer patients diagnosed with various types of malignancies, including breast, colorectal, esophageal, stomach, liver, lung, and pancreatic cancers. Additionally, 500 healthy controls (HCs) were included in the study to establish a comparative framework. The samples were systematically allocated into training and testing cohorts to optimize the diagnostic model. Remarkably, the results from the training, testing, and independent validation cohorts demonstrated impressive AUC values of 0.907, 0.908, and 0.868, respectively, indicating the robustness of the model in distinguishing between cancerous and non-cancerous states. Furthermore, specificity metrics in the testing and independent validation cohorts were recorded at 100% and 98.6%, respectively, with the training cohort exhibiting a specificity of 98%. These findings suggest that plasma methylation marker profiling via the TOTEM framework holds significant potential for precise identification and localization of multiple cancers, thereby advancing the field of oncological diagnostics and improving patient outcomes [83].

Principal component analysis (PCA) is a widely recognized technique in the field of data analysis, particularly esteemed for its capability to perform dimensionality reduction on high-dimensional datasets. This method operates by linearly transforming raw data into a set of linearly independent representations, effectively extracting the principal feature components that capture the most variance within the data [84]. Through this transformation, PCA aids in simplifying complex datasets while retaining essential information, thus facilitating better data visualization and interpretation. In contrast, partial least squares discriminant analysis (PLS-DA) is an adaptable analytical method that serves multiple purposes, including discriminative variable selection, descriptive modeling, and predictive modeling. PLS-DA is fundamentally a classification technique that exhibits significant similarities to a supervised application of PCA. It allows for the identification of the most relevant variables that contribute to the differentiation between predefined classes in the dataset, thereby enhancing classification accuracy [85]. Consequently, both PCA and PLS-DA are invaluable tools in the toolkit of data analysts and researchers, offering unique yet complementary approaches for handling complex data structures.

The application of t-distributed stochastic neighbor embedding (t-SNE) analysis, while a valuable tool for visualizing high-dimensional data, may inadvertently produce misleading clusters that can misguide researchers in their investigations [86]. In the realm of cancer research, particularly in the study of GIC, understanding molecular profiles is critical. Hierarchical clustering analysis serves as a robust method for delineating such profiles [87]. Another study focuses on the functional proteomic heterogeneity observed in esophageal squamous cell carcinoma (ESCA), stomach adenocarcinoma (STAD), colon adenocarcinoma (COAD), and rectal adenocarcinoma (READ), utilizing multiple analytical approaches, including PCA, PLS-DA, t-SNE, and hierarchical clustering. These methodologies collectively contribute to a comprehensive understanding of the distinct characteristics associated with these four types of GI malignancies. The findings underscore the potential of functional proteomic profiling to uncover candidate proteins that can aid in clinical diagnosis and prognostic evaluations, effectively revealing unique patterns across the various forms of GIC [88]. Further analysis of infrared spectral data obtained from serum samples was conducted through PCA, identifying specific spectral regions that exhibit notable differences in intensity between affected and healthy cohorts. Notably, the infrared spectral regions ranging from 3500 to 3000 cm^−1^, 1700 to 1600 cm^−1^, and 1090 to 1070 cm^−1^ were determined to possess significant diagnostic relevance for GC. The implementation of machine learning algorithms facilitated the distinction between GC patients (n = 96) and HCs (n = 96), achieving a sensitivity of 89.7% and a specificity of 87.2%. The AUC, assessed through receiver operating characteristic (ROC) analysis, was calculated to be 0.901 [89]. Furthermore, dietary patterns were investigated in relation to CRC risk, contrasting “unhealthy” diets, characterized by high meat and processed food consumption (Western dietary pattern, WDP), with “healthy” diets rich in fruits and vegetables (prudent dietary pattern, PDP). Utilizing PCA, the study revealed a correlation between dietary habits and CRC incidence. The results suggest that adherence to a prudent dietary pattern may significantly reduce the likelihood of developing CRC, whereas a Western dietary pattern appears to correlate with an elevated risk of the disease [90]. This multifaceted analysis highlights the intricate interplay between dietary choices and cancer risk, emphasizing the importance of both proteomic profiling and lifestyle factors in understanding and combating GIC.

GI carcinomas represent a heterogeneous group of tumors that affect various components of the GI tract and associated digestive organs, including the pancreas, small intestine, esophagus, colon, rectum, liver, and stomach. A significant area of research has focused on the role of small functional noncoding RNAs, particularly miRNAs, which engage in post-transcriptional regulation by binding to the 3′-untranslated regions (3′-UTRs) of target genes in a complementary manner. This regulatory mechanism significantly influences gene expression, underscoring the importance of miRNAs in both normal physiological processes and pathological conditions, including the onset and progression of cancer. Emerging evidence highlights the antioncogenic properties of miR-145 across various malignancies, including those originating in the GI tract. miR-145 has been implicated in critical biological processes related to cancer, such as angiogenesis, cellular proliferation, differentiation, carcinogenesis, apoptosis, metastasis, and treatment resistance [91]. To further elucidate the connections between miRNAs and various diseases, researchers have developed an ensemble learning framework known as ELMDA. This framework has demonstrated high accuracy in validating putative miRNAs associated with gastric, prostate, and colon neoplasms, achieving verification rates of 100%, 94%, and 90%, respectively, using the HMDD V3.2 database [92]. In addition to miRNAs, the expression levels of peptidylprolyl isomerase H (PPIH) have emerged as significant in the context of cancer prognosis. Studies indicate that mRNA and protein levels of PPIH are markedly elevated in patients with BC, COAD, and liver hepatocellular carcinoma (LIHC), correlating with poorer prognostic outcomes. Conversely, lower serum levels of PPIH were observed in patients with LIHC, COAD, BC, and GC. When combined with conventional tumor markers, these lower serum levels of PPIH have the potential to enhance diagnostic sensitivity and specificity. Furthermore, the secretion of PPIH by tumors suggests its role as a predictive biomarker for cancer, highlighting its possible involvement in the initiation and progression of malignancies. In conclusion, the interplay between miRNAs, particularly miR-145, and PPIH indicates their pivotal roles in the biological mechanisms underlying GI carcinomas [93]. Understanding these molecular interactions may pave the way for novel therapeutic strategies and improved diagnostic tools in the management of GIC.

Mendelian randomization (MR) employs genetic variation to explore causal questions regarding the influence of modifiable exposures on health outcomes. Building upon Mendel’s laws of heredity and instrumental variable estimation, MR allows for causal inferences even amidst unobserved confounding variables. This study investigates the association between height and cancer risk, the interplay between mental illness and digestive tract tumors, and the relationship between OSA and various GI disorders [94]. Research indicates a positive association between height and cancer incidence, primarily derived from studies conducted in Western populations. Utilizing data from the China Kadoorie Biobank (CKB) prospective cohort, significant increases in lymphoma (OR 1.18, 95% CI 1.04–1.34, *p* = 0.010), CRC (OR 1.09, 95% CI 1.02–1.16, *p* = 0.010), and GC (OR 1.07, 95% CI 1.00–1.14, *p* = 0.044) were noted with every 10 cm increase in height. MR analyses further confirmed a correlation between genetically predicted height and elevated risks of GC (OR 1.14, 95% CI 1.02–1.29, *p* = 0.0233) and lung cancer (LIHC) (OR 1.17, 95% CI 1.02–1.35, *p* = 0.0244) [95]. Exploring the genetic links between psychiatric conditions and digestive tract tumors, MR analysis of GWAS data investigated six mental illnesses—schizophrenia, bipolar disorder, major depressive disorder (MDD), attention deficit hyperactivity disorder, autism spectrum disorder, and panic disorder (PD)—in relation to three types of digestive tract cancers, namely esophageal cancer (EC), GC, and CRC. The analysis revealed no causal relationship between psychiatric disorders and the risks of GC or EC, nor did it establish psychiatric conditions as risk factors for CRC. Notably, PD was associated with a decreased risk of CRC (OR = 0.79, 95% CI 0.66–0.93; *p* = 0.01), suggesting a potential protective effect [96]. Another study also examined the genetic basis for GI disorders in individuals with obstructive sleep apnea (OSA) through MR analyses. A comprehensive assessment of 19 GI disorders, including diverticular disease, gastroesophageal reflux disease, UC, Crohn’s disease, chronic gastritis, irritable bowel syndrome, acute pancreatitis, nonalcoholic fatty liver disease, cirrhosis, bile duct calculus, gallbladder calculus, pancreatic cancer, GC, CRC, and EC, was conducted. The MR investigation provided strong evidence of an independent causal relationship between genetically predicted OSA and an increased risk of inflammation-related GI disorders, while no significant causal link was identified between OSA and malignancies [97]. MR serves as a valuable tool in elucidating the complex relationships between genetic factors and health outcomes. This analysis not only highlights the associations between height and cancer risk but also clarifies the lack of causal connections between mental illnesses and digestive tract tumors. Additionally, it underscores the independent causal relationship between OSA and certain GI disorders, paving the way for further research into the genetic underpinnings of these associations. Such insights can inform preventive measures and therapeutic strategies for managing these health conditions.

The researchers utilized a 384-well microtiter plate format (specifically, the white opaque OptiPlate™ from Revvity) to perform an amplified luminescent proximity homogeneous assay-linked immunosorbent assay (AlphaLISA). The assay involved the incorporation of 2.5 µL of serum diluted at a ratio of 1:100 with an equal volume of either GST or GST-KIAA0513 proteins (at a concentration of 10 µg/mL) in a specially prepared AlphaLISA buffer, which maintained a pH of 7.4 and comprised 0.1% casein, 0.5% Triton X-100, 1 mg/mL dextran-500, and 0.05% Proclin-300 (Revvity). Following a 6–8 h incubation period at room temperature, we proceeded to add anti-human IgG-conjugated acceptor beads (2.5 µL at 40 µg/mL) and glutathione-conjugated donor beads (2.5 µL at 40 µg/mL) to the reaction mixture. This combination was subsequently incubated in the dark at room temperature for a duration ranging from 1 to 14 days. The chemical emissions resulting from the assay were quantified using an EnSpire Alpha microplate reader (Revvity). The findings from the AlphaLISA revealed that patients diagnosed with acute ischemic stroke (AIS), diabetes mellitus (DM), CVD, obstructive sleep apnea syndrome (OSAS), chronic kidney disease (CKD), and various solid malignancies—including esophageal, gastric, colon, lung, and breast cancers—exhibited markedly elevated serum antibody levels directed against the recombinant KIAA0513 protein compared to healthy control subjects. These elevated serum anti-KIAA0513 antibody levels may serve as biomarkers indicative of prevalent arterial pathologies that contribute to both atherosclerosis and malignancy. Consequently, they appear to hold significant diagnostic potential for conditions, such as AIS, transient ischemic attack (TIA), DM, CVD, OSAS, CKD, and various solid tumors [98].

Recent immunohistochemical analyses have identified the expression of carcinoembryonic antigen-related cell adhesion molecule 6 (CEACAM6) on cellular surfaces, particularly in myeloid and epithelial cells. CEACAM6 belongs to the immunoglobulin superfamily and is implicated in various cellular processes that facilitate cancer progression. The expression of CEACAM6 has been associated with the prevention of apoptosis, the promotion of drug resistance, and enhanced cancer cell invasion and metastasis [99]. Numerous studies have demonstrated that CEACAM6 expression is markedly elevated in multiple types of cancer, including breast, colorectal, gastric, non-small cell lung, and pancreatic cancers. This overexpression correlates with increased tumor invasiveness and metastatic potential, indicating its significance in the cancer continuum. CEACAM6 appears to exert its effects through the activation of critical signaling pathways. It can engage the epidermal growth factor receptor (EGFR) or directly activate pathways such as ERK1/2/MAPK and SRC/focal adhesion kinase/PI3K/AKT. These signaling cascades are crucial for promoting tumor cell behaviors, such as invasion, migration, resistance to anoikis, and enhanced angiogenesis [100]. The evidence supporting the role of CEACAM6 in cancer biology underscores its potential as both a therapeutic target and a biomarker for various malignancies. As ongoing research continues to unravel the complexities of CEACAM6-mediated signaling, it may pave the way for innovative strategies in cancer treatment and management.

ICAM1 is a pivotal gene associated with the infiltration of immunological cells, particularly M1 macrophages and CD8+ T cells, in GC and CRC. Its expression levels have been linked to various immunological parameters, making it a promising candidate for a prognostic biomarker across a spectrum of cancer types. This exploration is critical, as it not only enhances our understanding of ICAM1’s role in carcinogenesis but also its potential impact on tumor immunity. Data were aggregated from several prominent databases, including HPA, cBioPortal, GTEx, CCLE, and TCGA, to ascertain the expression levels of ICAM1 in relation to tumor mutation burden and MSI in 11 different malignancies. Additionally, a detailed investigation was conducted to identify immunological features causally associated with GI tract cancers. The analysis indicated a significant association between ICAM1 expression levels and tumor mutation burden in 11 malignancies. Notably, in eight of these tumors, a correlation with MSI was also observed [101]. Furthermore, 78 distinct immunological features were identified as being causally linked to GI tract malignancies, encompassing cancers of the esophagus (12 traits), stomach (13 traits), small intestine (22 traits), colon (12 traits), and rectum (19 traits). Of these, 60 immunological features were discerned as protective variables against GI tract cancers, with specific traits linked to ESCA, GC, small intestine cancer, colon cancer, and rectal cancer. The findings underscore the multifaceted role of ICAM1 in cancer biology, particularly regarding its influence on tumor immunity and the involvement of immune cell infiltration in the TME. Notably, at least two studies have demonstrated a causal relationship between seven immunological characteristics, such as “CCR2 on CD14- CD16+ monocyte” and “CD19 on IgD+ CD38- naive”, and GI tract malignancies. These associations highlight the complexity of the immune landscape in cancer progression and the potential of ICAM1 as a prognostic biomarker [102]. ICAM1 plays a vital role in the interplay between tumor biology and immune response, presenting opportunities for novel therapeutic interventions and improved patient prognostication in various cancers. Further research is warranted to elucidate the mechanisms underlying these associations and to harness ICAM1’s potential in cancer therapy.

Recent studies have identified the stroma A reactive invasion front area (SARIFA) as a promising histomorphological biomarker with substantial predictive value for various malignancies, including pancreatic, colorectal, gastric, and prostate cancers. SARIFA is characterized by the absence of a stromal reaction and is marked by direct interactions between tumor cells and adipocytes. Its assessment through hematoxylin and eosin (H&E) slides, which are routinely available in clinical settings, has demonstrated high interobserver agreement, making it a practical tool for pathologists [103]. Moreover, SARIFA highlights a distinct tumor biology influenced by metabolic reprogramming. Tumor cells in SARIFA-positive tumors benefit from direct contact with adipocytes, which provide an external supply of lipids, thereby facilitating tumor growth and progression. An analysis of SARIFA status in tissue samples from the COAD and READ cohorts of the Cancer Genome Atlas (TCGA) involved a thorough investigation of 207 CRC patients. The findings revealed strong correlations between H&E-based SARIFA positivity and detrimental outcomes, including poor overall survival, disease-specific survival, and progression-free survival. Notably, these correlations were found to partially surpass the predictive capabilities of traditional prognostic variables. Interestingly, SARIFA positivity did not correlate with established high-risk genetic profiles, such as BRAF V600E mutations or microsatellite stability, suggesting that SARIFA may serve as an independent prognostic factor. Furthermore, SARIFA-positive CRCs exhibited transcriptional similarities with consensus molecular subtypes (CMS) 1 and 4 as recognized molecular classifications of CRC. They also displayed unique patterns of differential gene expression related to lipid metabolism and elevated stromal cell infiltration scores. The implications of SARIFA extend beyond prognostication; it also appears to influence treatment responses. SARIFA-positive CRC tumors demonstrated distinct responses to therapies, as predicted by gene expression-based drug sensitivity analyses [104]. These findings underscore the potential of SARIFA as both a prognostic biomarker and a therapeutic target in CRC, warranting further investigation into its role in clinical practice and treatment stratification.

In an effort to elucidate the taxonomic configurations associated with various cancers, a comprehensive study was conducted involving whole-genome shotgun sequencing (WGS) and gas chromatography/mass spectrometry (GC/MS) across distinct patient groups, specifically 40 patients with CRC, 45 with GC, 71 with BC, 34 with LIHC, 50 with melanoma, 60 with lymphoid neoplasms, and 40 with acute myeloid leukemia (AML). The microbiome data from these patients were meticulously compared to those of age- and sex-matched HCs. Notably, a significant reduction in the gut microbiome cancer index was observed in all patient groups, with the exception of those with AML. Furthermore, a detailed analysis revealed that the abundances of seven species of *Faecalibacterium* exhibited a negative correlation with most amino acids and formic acid, while demonstrating a positive correlation with the levels of short-chain fatty acids, specifically acetic, propanoic, and butanoic acid. Among the metabolites examined, the relative concentration of formic acid was found to be significantly elevated in all case groups compared to HCs. The findings from this research underscore the potential for novel diagnostic and predictive methodologies aimed at evaluating intestinal dysbiosis in cancer patients. The release of these data provides a substantial foundation for further exploration into the microbiome’s role in cancer pathogenesis and its utility as a biomarker for clinical assessment and intervention strategies [105].

Table 3 presents a comprehensive overview of potential biomarkers that may play a significant role in the study of synchronous gastric and colon cancers. These biomarkers are crucial for understanding the pathophysiological mechanisms underlying the co-occurrence of these two malignancies, as well as for developing targeted therapeutic strategies. The identification and validation of such biomarkers could enhance diagnostic accuracy, allow for better prognostic assessments, and facilitate personalized treatment approaches. By elucidating the molecular profiles associated with synchronous gastric and colon cancers, this research aims to contribute to the growing body of knowledge in oncological biomarker discovery and ultimately improve patient outcomes.

## 7. Conclusions

The co-occurrence of GC and CRC is a phenomenon that is gaining recognition in contemporary medical research. The increasing prevalence of these dual syndromes necessitates a thorough genetic exploration to enhance patient care and treatment outcomes. Among the various genetic factors under investigation, the KRAS gene, Wnt signaling pathway, and polymorphisms in nucleic acids and chemokine ligands stand out as the most extensively studied components. Despite the burgeoning interest in the role of natural plant extracts in cancer therapy, research in this area remains nascent. Nevertheless, the volume of studies focusing on potential biomarkers for the detection of the association between GC and CRC has seen a marked increase. This growing body of work underscores the importance of interdisciplinary approaches in unraveling the complexities of these malignancies and highlights the need for continued exploration into genetic and biochemical markers that may facilitate early diagnosis and improved management of affected patients.

## Figures and Tables

**Table 1:** Main syndromes associated with GC and CRC.

	The Frequency of Their Occurrence	Related Genes	Cancer Risk Estimates	Surveillance	Management Guidelines	References
LFS	80% carriers of a germline TP53 mutation at age 70	TP53 encodes p53	Males > 70%, females > 90%	Earlier discovery of TP53 mutant carriers results in reduced morbidity and improved survival	Clinical therapy varies and should be divided into classes according to the kind of mutation	[15,16,17,18]
LS	Between 5 and 10% of CRC cases are hereditary. The majority are associated with LS	MMR	Risk of CRC is 80%, risk of endometrial cancer is 60%	The most common malignancies are: - CRC and GC in men - Endometrial cancer in women	Chemotherapy, immunotherapy, and vaccines	[19,20,21,22]
FAP	A prevalence of between 1:20,000 and 1:10,000 in Western countries and 1:17,400 in Japan	APC	Incidence rates in 50-year-old individuals with FAP were 22.8% and 7.6%, respectively	Poor compliance	Colonectomy and multidisciplinary and individualized approaches	[23,24,25,26]
JPS	An estimated incidence varying from 1:16,000 to 1:100,000	SMAD4 or BMPR1A	75% of cases have an autosomal-dominant genetic disease	A low risk of carcinogenesis	No curative treatment	[27,28,29]
PJS	PJS is a rare hereditary syndrome - 8/30 (27%) patients in the Russian population	STK11	Patients without STK11 mutations may have had a decreased risk of developing cancer and later start of symptoms	Due of the problems of polyps, children with PJS are at a significant risk of requiring multiple laparotomies	Given the diffuse involvement of the gut, substantial intestinal resection and early surgical decision-making are not recommended	[30,31,32,33]
CAC	CAC is the leading cause of death among long-standing IBD patients	p53 mutations	The first step in the development of serrated CRC is known as GM	The considerable proportion that appears to originate from non-adenomatous-looking mucosa that displays rapid cancer progression but fails to reveal neoplasia on biopsy	STING	[34,35,36,37,38]
GISTs	An incidence of 7–15 cases per million	PDGFRA D842V mutation	The stomach (n = 7, 35%), colon (n = 3, 15%), and peritoneum (n = 1, n = 5%) were the most commonly affected sites (n = 9, 45%)	The long-term prognosis is strongly correlated with the size of the tumor and the number of mitoses	Targeted therapy type, [18F] FDG PET/CT characteristics, recurring metastatic GISTs, and the nomogram could produce more accurate responses	[39,40,41]

**Table 2:** Natural products as possible treatment options.

Natural Products	Biological Activities	References
Arctiin	- Antibacterial, antitumor, antioxidative, antiproliferative, and antisenescence effects	[71]
	- Anticancer activity is mediated by its ability to target multiple cellular processes, thus impeding tumor growth and development	[72]
Noscapine	- Anticancer properties	[73]
	- Targets and eliminates cancer cells through apoptosis	[74]
Guggulsterone	- Potential to disrupt the mechanisms that confer resilience to cancer cells	[75]
	- Diminishes the viability of certain cancer cells	[76]
Resveratrol	- Potential protective effects against various cancers and cardiovascular diseases	[77]
	- Anti-inflammatory and anticancer properties, which are mediated through the regulation of lncRNAs	[78]
Garcinol	- Notable antioxidant, anti-inflammatory, and HAT inhibition properties, alongside its role as an miRNA deregulator	[79]
	- Inhibits metastasis, induces apoptosis, and targets pivotal molecular pathways involved in cancer progression	[80]
Minerals(trace elements)	- Zinc enhances immune system functionality and facilitates wound healing - Selenium is recognized for its antioxidant properties	[81]
Vitamins	- Have the potential to significantly lower cancer risk by neutralizing free radicals that can lead to cellular damage	[82]

**Table 3:** Potential biomarkers in the study of synchronous gastric and colon cancers.

Biomarkers	Utilizations	References
TOTEM	- In the identification and tracking of cancer signals through methylation markers	[83]
PCA	- In the field of data analysis, particularly esteemed for its capability to perform dimensionality reduction on high-dimensional datasets	[84]
PLS-DA	- In discriminative variable selection, descriptive modeling, and predictive modeling	[85]
t-SNE	- Used for visualizing high-dimensional data, although it may inadvertently produce misleading clusters that can misguide researchers in their investigations	[86]
Hierarchical clustering analysis	- A robust method for delineating molecular profiles of cancer	[87]
miRNAs	- In critical biological processes related to cancer, such as angiogenesis, cellular proliferation, differentiation, carcinogenesis, apoptosis, metastasis, and treatment resistance	[91,92,93]
MR	- Employs genetic variation to explore causal questions regarding the influence of modifiable exposures on health outcomes.	[94,95,96,97]
AlphaLISA	- Elevated serum of anti-KIAA0513 antibody levels may serve as biomarkers indicative of prevalent arterial pathologies that contribute to both atherosclerosis and malignancy	[98]
CEACAM6	- Has been associated with the prevention of apoptosis, the promotion of drug resistance, and enhanced cancer cell invasion and metastasis	[99,100]
ICAM1	- In cancer biology, particularly regarding its influence on tumor immunity and the involvement of immune cell infiltration in the TME	[101,102]
SARIFA	- Is characterized by the absence of a stromal reaction and is marked by direct interactions between tumor cells and adipocytes	[103,104]
microbiome	- In evaluating intestinal dysbiosis in cancer patients	[105]

## Data Availability

Not applicable.
